# Food Preference and Appetite after Switching between Sweet and Savoury Odours in Women

**DOI:** 10.1371/journal.pone.0146652

**Published:** 2016-01-11

**Authors:** Mariëlle G. Ramaekers, Pieternel A. Luning, Catriona M. M. Lakemond, Martinus A. J. S. van Boekel, Gerrit Gort, Sanne Boesveldt

**Affiliations:** 1 Food Quality and Design, Wageningen University, PO Box 17, 6700 AA Wageningen, The Netherlands; 2 Biometris, Wageningen University and Research Centre, PO Box 100, 6700 AC Wageningen, The Netherlands; 3 Division of Human Nutrition, Wageningen University, PO Box 8129, 6700 EV Wageningen, The Netherlands; Barnard College, Columbia University, UNITED STATES

## Abstract

**Background:**

Exposure to food odours increases the appetite for congruent foods and decreases the appetite for incongruent foods. However, the effect of exposure to a variety of food odours, as often occurs in daily life, is unknown.

**Objective:**

Investigate how switching between sweet and savoury odours affects the appetite for sweet and savoury products.

**Design:**

Thirty women (age: 18-45y; BMI: 18.5-25kg/m^2^) intensely smelled the contents of cups filled with banana, meat or water (no-odour) in a within-subject design with four combinations: no-odour/banana, no-odour/meat, meat/banana and banana/meat. Participants received one combination per test day. In each combination, two cups with different fillings were smelled for five minutes after each other. Treatment order was balanced as much as possible. The effects of previous exposure and current odour on the appetite for (in)congruent sweet and savoury products, and odour pleasantness were analysed. A change from meat to banana odour or banana to meat odour was referred to as switch, whereas a change from no-odour to meat odour or no-odour to banana odour was no-switch.

**Results:**

The current odour (P<0.001), as opposed to the previous exposure (P = 0.71), determined the appetite for (in)congruent sweet and savoury products, already one minute after a switch between sweet and savoury odours. The pleasantness of the odour decreased during odour exposure (P = 0.005).

**Conclusions:**

After a switch, the appetite for specific products quickly adjusted to the new odour and followed the typical pattern as found during odour exposure in previous studies. Interestingly, the appetite for the smelled food remained elevated during odour exposure, known as sensory-specific appetite, whereas the pleasantness of the odour decreased over time, previously termed olfactory sensory-specific satiety. This seeming contradiction may result from different mechanisms underlying the odour-induced anticipation of food intake versus the decrease in hedonic value during prolonged sensory stimulation.

## Introduction

Unhealthy eating habits such as unhealthy food choices or overeating increase the prevalence of obesity, diabetes, cancer, cardiovascular and other diseases [1–3]. Therefore, it is important to understand how various factors, for example sensory processes, influence our eating behaviour. Sensory processes play a role in food selection in several ways. First, associations between the nutrient composition and the sensory properties of foods, such as appearance, smell and taste, are formed due to repeated exposure in our daily lives [4]. These associations partly determine the pleasure that is derived from foods [5,6], whereupon pleasantness influences food selection. Moreover, these associations also facilitate the estimation of the nutrient composition of foods based on the sensory properties [4] and this information can be used for food selection, for example in case of nutrient deficits [7,8]. Furthermore, recently consumed foods modulate food preference, which is likely driven by the need for variety. For example, the preference for savoury products decreases after eating a savoury meal, a phenomenon referred to as sensory-specific satiety [9,10]. Finally, external factors such as exposure to sight, taste or smell of foods change our food preference [11–15]. It has been widely demonstrated that exposure to food cues increases the preference for the cued food [14–20]. For example, sweet odours increased the appetite for sweet products and savoury odours the appetite for savoury products [17,18]. Moreover, sweet odours also decreased the appetite for savoury products and savoury odours for sweet products [17,18].

The precise mechanism behind these findings has not yet been elucidated, although we have proposed that this increase in appetite for congruent foods and decrease in appetite for incongruent foods may be caused by cephalic phase responses [17]. Cephalic phase responses prepare the body for the intake and digestion of foods [21–23] and are elicited by food odours and other food cues. In general, sweetness is associated with sugar content and savouriness with protein content [4,7], with distinct routes of digestion for different macronutrients. Therefore, determining the type of food by exposure to food cues before ingestion, may prepare the body for the digestion of the specific macronutrients of the anticipated foods [17,24]. It may be that once the body is prepared for the intake of a food with a certain (macro)nutrient composition, it is less favourable to ingest a food with a very different (macro)nutrient composition [17].

In daily life though, for example by strolling through a (super)market, exposure to a variety of food cues that prime for a wide variety of foods, may induce confusion in the body. Previous exposures to food cues may possibly interfere with exposures to new food cues. If our body indeed specifically prepares for the intake of cued foods, then it may take some time to switch the appetite for (in)congruent foods according to the characteristics of new food cues.

To our knowledge, the effect of switching between different food cues on general appetite and food preference has not been investigated before. The objective of the current study was to determine how switching between sweet and savoury food odours affects the appetite for sweet and savoury products, food preference and general appetite. General appetite and the appetite for sweet and savoury products were measured at several time points during odour exposure to explore if possible changes after switching are immediate or take time. The results could provide insight in the processes behind the effect of food cues on food preference in real life.

## Materials and Methods

### Participants

**Fig 1:**
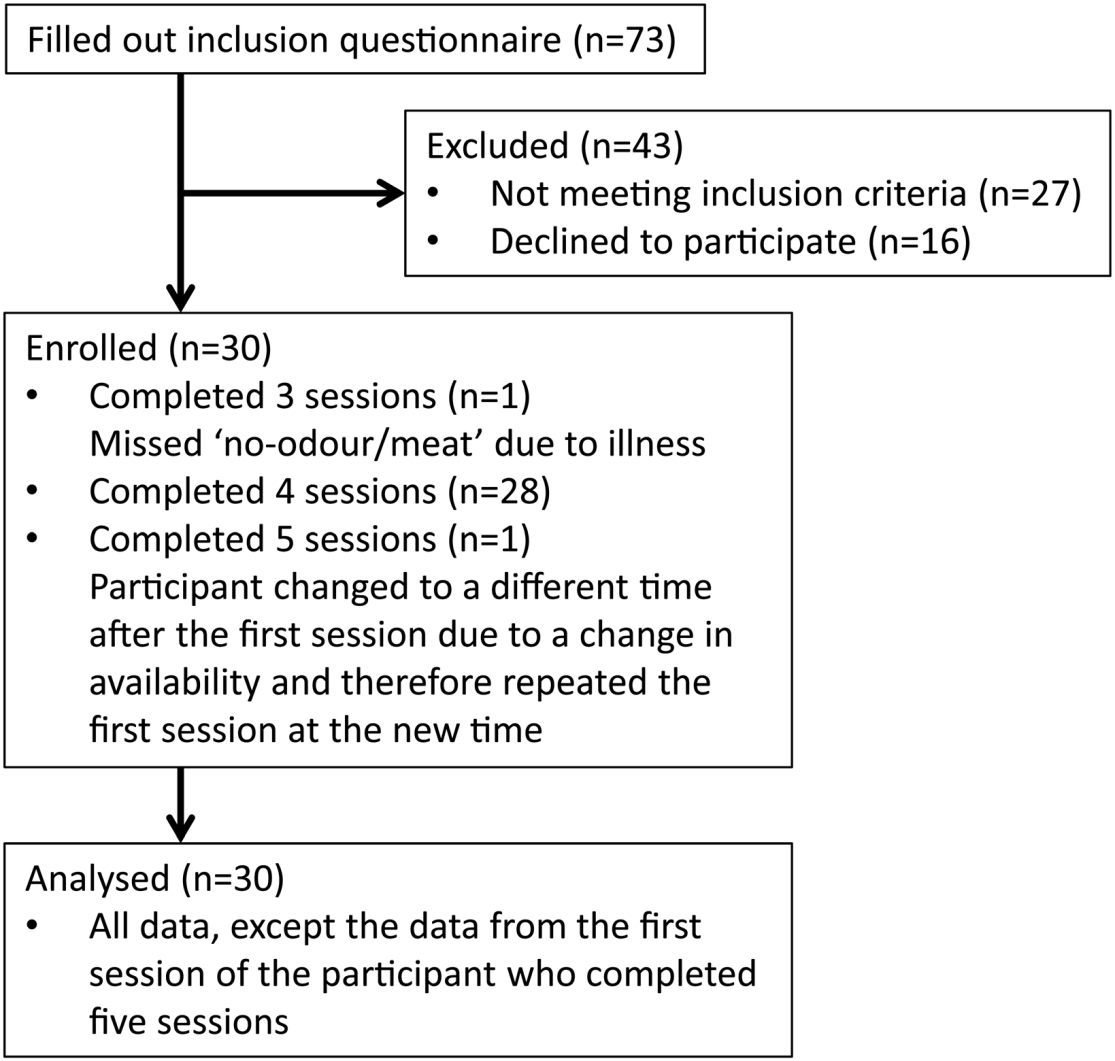
Flowchart that shows the participant enrolment in this study.

Thirty healthy women aged 18–45 (21.6±4.7) y and BMI 18.5–25 (21.9±1.3) kg/m^2^ were recruited from Wageningen and surroundings between 7 and 14 February 2013, by using an e-mail list with potential participants that was set-up by the Division of Human Nutrition, Wageningen University (Fig 1). Exclusion criteria were: dislike for banana, banana pie (Dutch pastry), steamed meat or beef soup (score < 5 on a nine-point scale), smoking, pregnancy or breast feeding during the last six months, lack of appetite, following an energy-restricted diet or change in body weight>5 kg during the last 2 months, hypersensitivity to any of the foods under study or being a vegetarian. It was explained to the participants that the influence of sensory signals on food choice was investigated. After the study, the participants were informed about the full study objectives. Twenty-nine participants completed the study and one participant missed the last session (no-odour/meat) due to illness. All participants signed an informed consent form before participation. All procedures were in accordance with the Helsinki Declaration of 1975 (as revised in 1983). This study was exempt from approval by the Medical Ethical Committee of Wageningen University, because the rules of conduct in the present research were not interventionist, with very little burden to the participants. This study was registered at the Dutch trial register (NTR3830; www.trialregister.nl/trialreg/admin/rctview.asp?TC=3830). The participants received a compensation of 25 euro.

### Experimental design

**Fig 2:**
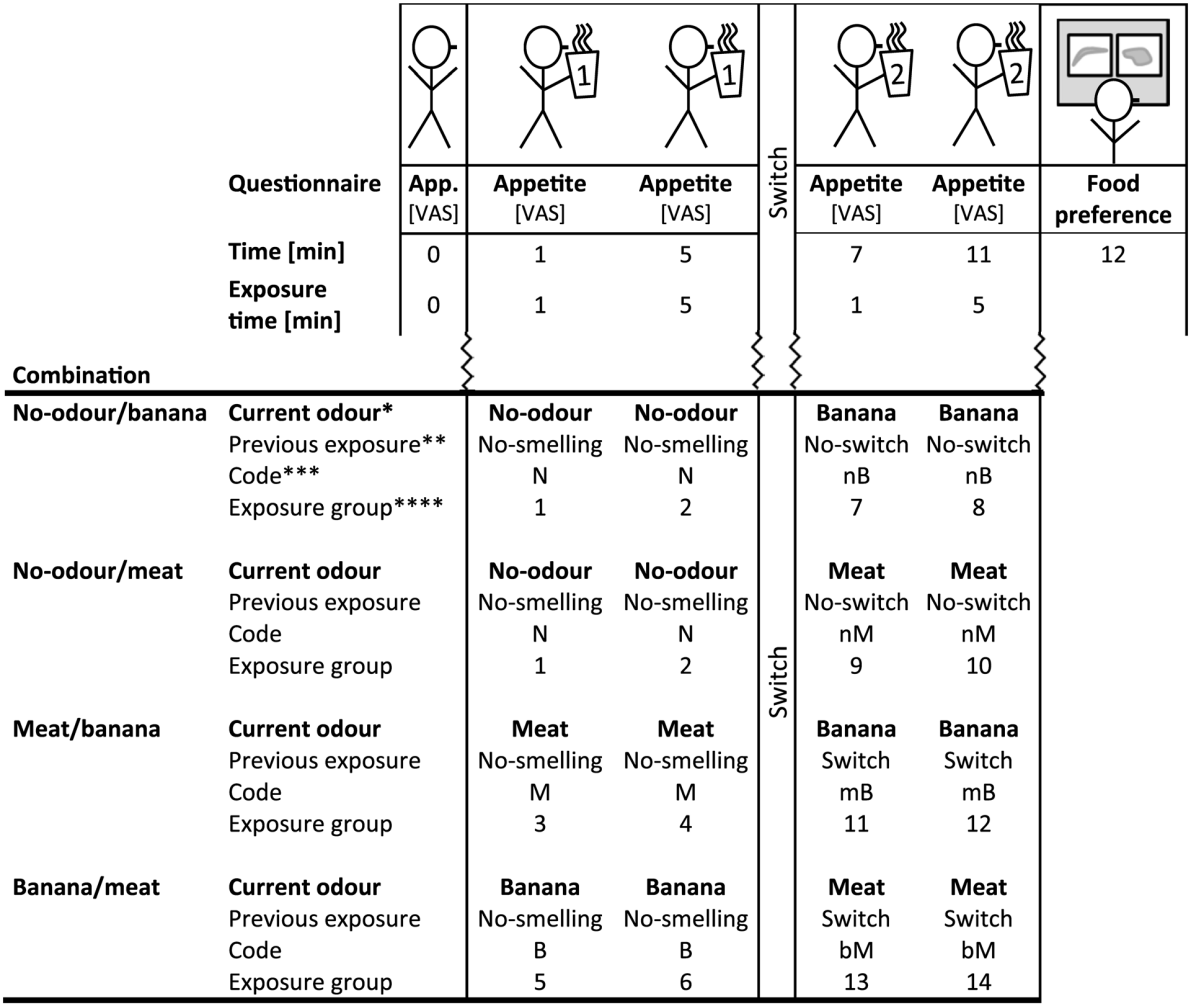
Schematic representation of the experimental design including explanation of the terminology used in the data analysis and result sections. * Current odour is presently smelled by the participants, which can be either the first or the second odour in a combination. ** Previous exposure is the exposure that preceded the current odour. *** Code is used in the results section and defines the combination of previous exposure and current odour. **** Exposure group is used for the data-analysis.

The participants intensely smelled the contents of cups filled with banana, meat or water (no-odour) in a within-subject design with four combinations: no-odour/banana, no-odour/meat, meat/banana and banana/meat (Fig 2). Each participant was exposed to one combination with two successive odour exposures and a one-minute break in between exposures, on each test day. These four combinations were set up to compare the appetite for specific products after a switch with the appetite for specific products after no-switch (no-odour as control). For example, to compare the appetite for banana, during smelling banana after smelling no-odour (no-switch), with the appetite for banana, during smelling banana after smelling meat (switch). We used no-odour/banana and no-odour/meat as controls instead of banana/banana and meat/meat, to exclude undesired differences in adaptation or specific satiation due to differences in exposure time to a specific odour. Each odour was smelled intensely for five minutes. Appetite measurements were taken at 1 and 5 minutes during exposure to the first odour and at 7 and 11 minutes during exposure to the second odour. The order of the combinations was balanced over the participants, and as much as possible over the test days. Each participant was scheduled on four separate days, preferably once per week at the same time of the day between 11.20 h and 13.40 h. The effects of the previous exposure and the current odour on the appetites for (in)congruent sweet and savoury products, food preference, general appetite and odour pleasantness were analysed. A change from meat to banana odour or from banana to meat odour was referred to as switch between odours, whereas a change from no-odour to meat odour or no-odour to banana odour was no-switch (Fig 2).

### Odours

The participants smelled the contents of cups that contained either 10 g of water (no-odour), a tablespoon of medium ripe mashed banana (banana odour) or a table spoon of warm steamed meat (stoofvlees, Coertjens, Belgium; meat odour). A tissue and a plastic lid were placed over the filling to prevent visual cues and odour contamination in the sensory room.

### Standardizing hunger state

The visits of each participant were scheduled at the same time of the day to standardize the individual hunger state. On the first test day, participants were instructed to consume a normal amount of breakfast, at least 2.5 hours before the start of the experiment. After this time, only water and weak tea were allowed. On the remaining test days, the participants were requested to consume the same breakfast as on the first test day. The diaries in which participants recorded their breakfast, changes in physical activity and health problems were checked for possible confounders and to increase commitment to the study rules.

### Procedure

Upon the first arrival, the participants were informed on the procedure, including a demonstration of how to smell intensely by placing the nose above the cup and breathing through the nose. The participants then settled down in sensory booths with overpressure (Biotechnion of Wageningen University) with two cups in front of them. Instructions were given on a computer screen (E-prime, v2.0). Participants first filled out the appetite questionnaire at baseline (t = 0). Subsequently, they were requested to remove the plastic lid from the first cup, while keeping the tissue on the cup, and then intensely smell the content of the first cup. The appetite questionnaire was repeated 1 and 5 minutes after the start of the smelling. The participants were encouraged by the text on the computer to continue intense smelling during the whole five minutes of odour exposure. After five minutes, the participants placed back the lid on the first cup and had a one-minute break. Subsequently, they followed the same procedure with the second cup and filled out the appetite questionnaire at t = 7 and 11 minutes. After five minutes of intensely smelling the content of the second cup, participants placed back the lid and filled out the food preference questionnaire. At the end of the session, the participants had to choose between a banana and a bread roll with steamed meat that were placed on a plate in front of them. The participants were asked about their thoughts on the study objective at the end of the study in an end evaluation.

### Measurements

An appetite questionnaire and food preference questionnaire were filled out during the experiment.

The appetite questionnaire measured hunger and desire-to-eat over time on 100 mm computerized visual analogue scales (VAS, not at all–very) [25]. Besides ‘general’ appetite, the appetite for 15 individual products was measured on 100 mm VAS in a randomised order (for example, ‘How large is your appetite for a banana at this moment?; not at all–very) [25]. These products were divided into banana, meat, sweet, savoury and staple products. Banana and banana pie were selected as banana products and bread roll with steamed meat and beef soup as meat products. Sweet products were mango, sweet pastry ‘tompouce’, strawberry yoghurt and M&M’s. Savoury products were bread roll with egg, tomato soup, cheese and salted peanuts. Staple products were bread bun, croissant and pancake. In addition, odour intensity (100 mm VAS, not at all–very) and feeling well (100 mm VAS, not at all–very) were added to the appetite questionnaire to check if the odours became overwhelming or affected the participants’ well-being. Finally, odour pleasantness was monitored over time (100 mm VAS, not at all–very).

The food preference questionnaire (FPQ) was a computerized task as previously used in Ramaekers *et al*. [17] and based on work of Finlayson *et al*. [26,27] (E-prime, v2.0; Psychology software tools, Sharpsburg, PA, USA) measuring food preference at the end of the 10 minutes of exposure to the odours. On each trial, two foods were simultaneously shown on a computer screen using digital colour photographs. The participants were asked to choose the food that they would like to eat most at that moment. The foods on the photographs were the same as the foods in the appetite questionnaire, plus additionally banana sweets, and little snack sausages. All banana products were compared against all non-banana products, including meat products. In addition, all meat products were compared against all non-meat products, leading to 84 comparisons. The frequency of each chosen product was determined.

### Data analysis

All variables were analysed with mixed linear models to account for the correlations between the repeated measures (SAS version 9.1.3; SAS Institute Inc., Cary, NC), where the fixed part of the model captures the treatment structure and the random part the correlations among observations [28]. All degrees of freedom were calculated according to the method by Kenward and Roger [29]. P-values < 0.05 (two-sided) were considered significant.

All figures show mean values ± SD of the raw data. Results in the text are estimated means ± SE, using a mixed model. Results in the text on transformed data were back-transformed to the original scale to facilitate interpretation.

#### Appetite questionnaire

Participants received four combinations of odours, but the data from the appetite questionnaire were not compared between combinations. Instead, we studied the effects of previous exposure (no-smelling, no-switch, switch), current odour (no-odour, banana, meat), exposure time (exposure time to the current odour, either 1 or 5 min; Fig 2) and their interactions on the following variables: general appetite, odour pleasantness, odour intensity, feeling well and change in appetite for specific products.

We defined a factor ‘exposure group’ as a combination of previous exposure, current odour and time (1, 5, 7, 11 min), because not all possible 3x3x4 combinations existed. ‘Exposure group’ represents the 14 actual combinations (Fig 2). We compared group means of ‘exposure group’ by using contrasts to analyse the effects of previous exposure (exposure groups 3+4 vs 7+8+9+10 vs 11+12+13+14), current odour (1+2 vs 3+4+9+10+13+14 vs 5+6+7+8+11+12), exposure time to the current odour (1 vs 5 min and 7 vs 11 min; exposure groups 1+3+5+7+9+11+13 vs 2+4+6+8+10+12+14), product and their interactions. To investigate whether previous exposure had an effect, B, nB and mB were compared against each other (exposure groups 5+6 vs 7+8 vs 11+12) and M, nM and bM were compared against each other (exposure groups 3+4 vs 9+10 vs 13+14; see Fig 2 for the codes). In case the effect of previous exposure was not significant, the data from B, nB and mB were grouped as banana odour (exposure groups 5+6+7+8+11+12), M, nM and bM as meat odour (exposure groups 3+4+9+10+13+14) and N as no-odour (exposure groups 1+2). When there was a significant effect of previous exposure, only the ratings at time 1 and 5 min were used for analysis of the effect of current odour (exposure groups 1+2 vs 3+4 vs 5+6).

**General appetite, odour pleasantness, odour intensity and feeling well:** Before statistical analysis, general appetite was calculated as the average of the hunger and the desire-to-eat scores. Subsequently, ratings for general appetite, odour pleasantness, odour intensity and feeling well were logit transformed, using ln((y/100+0.01)/(1-y/100+0.01)) to stabilize the variance. The fixed part of the mixed model consisted of the factor ‘exposure group’. Furthermore, general appetite at time = 0 was used as covariate for general appetite. The random part of the mixed models consisted of random effects for sessions and participants. For general appetite, we used an autoregressive order-1 correlation matrix for the correlations among repeated measurements at 1, 5, 7 and 11 min. For variables odour pleasantness, odour intensity and feeling well a compound symmetry correlation matrix was used. Correlations and residual variances were allowed to differ between current odours for odour pleasantness and odour intensity.

**Change in appetite for specific products:** Before statistical analysis, the mean change in appetite for banana products was calculated by averaging the change scores (current ratings minus ratings at time = 0) of the ratings of the appetite for banana and banana pie. Similarly, the ratings of the appetite for meat, sweet, savoury and staple products were averaged separately over the respective products. The fixed part of the mixed model consisted of the factor ‘exposure group’ (Fig 2), an extra factor ‘product’ (banana, meat, sweet, savoury, staple) and the interaction of ‘product’ with ‘exposure group’. The random part of the mixed model consisted of random effects for sessions and participants and an autoregressive order-1 correlation matrix for the correlations among repeated measurements at 1, 5, 7 and 11 min. Additionally, an unstructured covariance matrix was added to allow for unequal (co)variances among product groups per person and time. Correlations and residual variances were allowed to differ between current odours.

#### Food preference questionnaire (FPQ)

The effect of combination (no-odour/banana, no-odour/meat, meat/banana and banana/meat) on food preference was investigated using the FPQ. The food preference data were transformed using arcsine(sqrt(frequency/max)), with ‘max’ representing the maximum number of times a product could be chosen in a set, to stabilize variances. All comparisons between products in the FPQ were split into seven sets, with each set containing comparisons of two types of products. For example, the ‘banana-staple’ set contains the comparisons: banana vs bread bun, banana vs croissant, banana vs pancake, banana pie vs bread bun, banana pie vs croissant, banana pie vs pancake, banana sweets vs bread bun, banana sweets vs croissant, banana sweets vs pancake. Fixed effect factors were combination, set and their interaction. An unstructured covariance matrix specified the (co)variances between sets. Per set, we were interested in comparisons between combinations.

## Results

### Appetite for banana, meat, sweet, savoury and staple products

**Fig 3:**
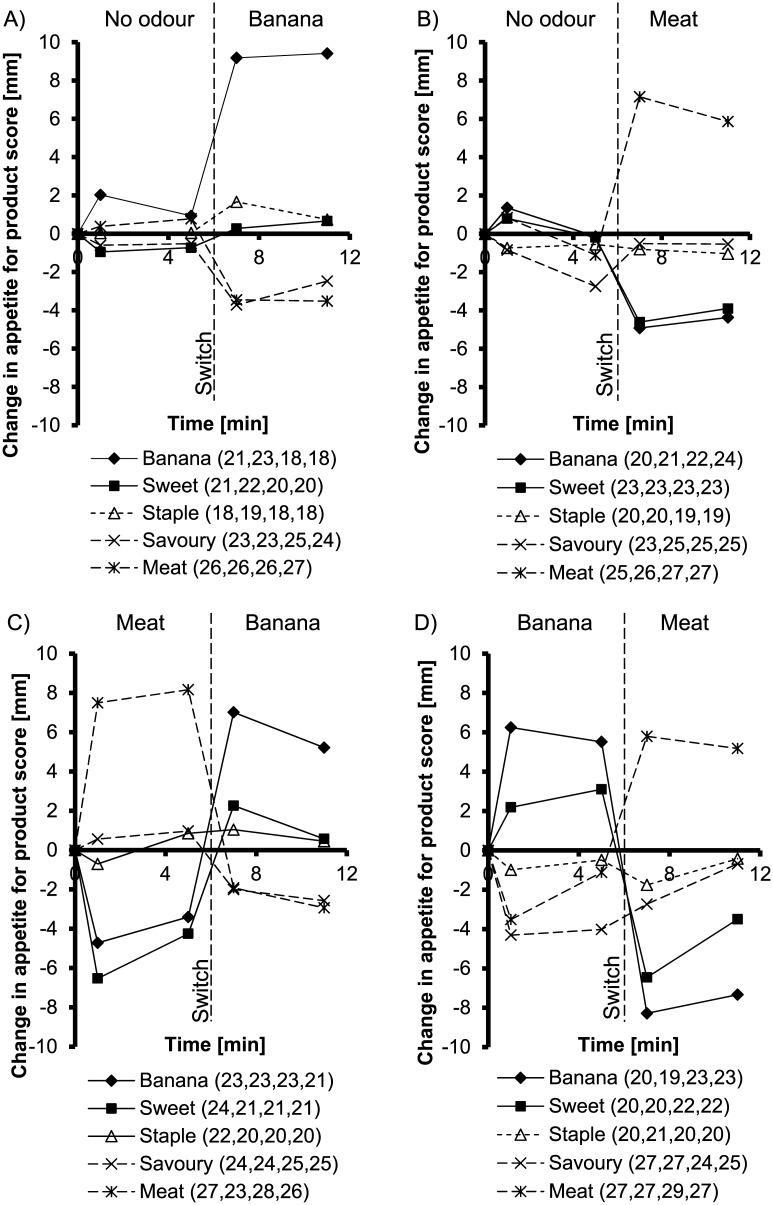
Mean change in appetite for banana, meat, staple, sweet and savoury products over time. Scores during exposure to **(A)** no-odour/banana, **(B)** no-odour/meat, **(C)** meat/banana and **(D)** banana/meat, measured on 100 mm VAS. The numbers between the brackets represent the SD at respectively 1, 5, 7 and 11 minutes. The dashed line represents the switch between odours.

Fig 3 shows the average changes in the appetites for banana, meat, sweet, savoury and staple products, further on named as the appetite for specific products. Previous exposure and its interactions with exposure time, current odour or product did not significantly affect the appetite for specific products (all P>0.05; Table 1). Therefore, all ratings were grouped per current odour (banana, meat and no-odour). The interaction between current odour and product was significant (P<0.001): the changes in the appetites for banana, meat, sweet, savoury and staple products did not differ among each other in the no-odour combination (P = 0.13), but differed during exposure to banana (P<0.001) and meat (P<0.001) odour. Exposure to banana odour increased the appetite for banana products (P<0.001), decreased the appetite for meat (P = 0.026) and savoury (P = 0.028) products and had no significant effect on sweet (P = 0.10) and staple (P = 0.48) products, compared with no-odour. Exposure to meat odour increased the appetite for meat (P<0.001) products, decreased the appetite for banana (P<0.001) and sweet (P<0.001) products and had no effect on savoury (P = 0.46) and staple products (P = 0.72), compared with no-odour.

**Table 1:** F-values with degrees of freedom and P-values of all factors with fixed effects for the change in appetite for specific products.

	Change in appetite for products	
**Fixed effects**		
Exposure time	F_2,336_ = 0.6	P = 0.55
Previous exposure	F_4,281_ = 0.8	P = 0.54
Current odour	F_2,303_ = 4.0	P = 0.019
Product	F_4,254_ = 9.6	P<0.001
ExpTime*Previous	F_4,298_ = 1.2	P = 0.32
ExpTime*Current	F_2,279_ = 2.6	P = 0.073
ExpTime*Product	F_8,473_ = 1.1	P = 0.38
Previous*Current	F_2,258_ = 0.7	P = 0.50
Previous*Product	F_16,502_ = 0.80	P = 0.71
Current*Product	F_8,445_ = 16	P<0.001
All 3- or 4-way interactions		P>0.20

### Preference for banana, meat, sweet, savoury and staple products, measured with FPQ

The preference of 84 pairs of food pictures was assessed, comparing banana and meat products against each other and against savoury, sweet and staple products (Table 2). When sets of banana and meat products were offered, the banana products were chosen more often when the last smelled odour was banana, than when the last smelled odour was meat (all P<0.05). In the banana-savoury and banana-staple sets, the banana products were more often chosen after the no-odour/banana combination, than after combinations no-odour/meat and banana/meat (all P<0.05). In the meat-sweet set, the meat products were chosen less often after combination no-odour/banana, than after combinations no-odour/meat and banana/meat (all P<0.05). The assessment of the 84 pairs took 3 to 4 min per session.

**Table 2:** Mean percentage of times a product was chosen per set of products, after exposure to different combinations, measured with the food preference questionnaire.

	Set						
Product 1	Banana-	Banana-	Banana-	Banana-	Meat-	Meat-	Meat-
Product 2	Meat*	Savoury*	Sweet*	Staple*	Savoury^#^	Sweet^#^	Staple^#^
Nr of comparisons per set	18	12	12	9	12	12	9
**No-odour/banana**	62^a^	60^a^	43	48^a^	46	33^a^	33
**Meat/banana**	58^a^	50^ab^	42	40^ab^	47	41^ab^	33
**No-odour/meat**	42^b^	47^b^	38	33^b^	53	56^b^	44
**Banana/meat**	42^b^	44^b^	43	33^b^	54	54^b^	46

Superscript with different letters denote significant differences at P<0.05

*Mean percentage of times that a banana product was chosen

^#^ Mean percentage of times that a meat product was chosen

### Actual food choice

After the odour exposure, thirteen participants always chose a banana and nine participants always chose a bread bun with steamed meat. Eight out of 30 participants switched their food choice between sessions. The choice for banana vs bread roll steamed meat was as follows: 19 vs 11 in no-odour/banana, 19 vs 11 in meat/banana, 12 vs 17 in no-odour/meat and 17 vs 13 in banana/meat.

### General appetite

General appetite ratings were on average 79 mm. There were no significant differences in general appetite between the four combinations at time = 0 min (F_3,83.4_ = 1.8; P = 0.16). General appetite ratings at times 1, 5, 7 and 11 were strongly related to the appetite ratings at time = 0 (F_1,124_ = 250; P<0.001; Table 3). General appetite did not change significantly over time (P = 0.52). The effect of previous exposure on general appetite was borderline significant (P = 0.08) and therefore, the effect of odour was investigated only at times 1 and 5 min. There were no significant differences between meat, banana and no-odour at times 1 and 5 min (M vs B vs N; P = 0.25). The interaction between previous exposure and current odour was not significant (F_2,167_ = 1.2; P = 0.31).

**Table 3:** F-values with degrees of freedom and P-values of all factors with fixed effects.

	General appetite		Odour pleasantness		Odour intensity		Feeling well	
**Fixed effects**								
Exposure time	F_2,327_ = 0.7	P = 0.52	F_2,190_ = 4.4	P = 0.013	F_2,185_ = 47	P<0.001	F_2,346_ = 0.4	P = 0.70
Previous exposure	F_4,196_ = 2.1	P = 0.079	F_4,68.4_ = 0.3	P = 0.88	F_4,91.5_ = 0.7	P = 0.60	F_4,206_ = 0.6	P = 0.68
Current odour	F_2,150_ = 1.4	P = 0.25^#^	F_2,98_ = 47	P<0.001	F_2,107_ = 103	P<0.001	F_2,230_ = 6.0	P = 0.003
ExpTime*Previous	F_4,324_ = 0.7	P = 0.58	F_4,128_ = 0.4	P = 0.79	F_4,128_ = 1.0	P = 0.40	F_4,346_ = 2.0	P = 0.097
ExpTime*Current	F_2,321_ = 0.8	P = 0.47	F_2,152_ = 3.5	P = 0.033	F_2,146_ = 7.7	P<0.001	F_2,346_ = 1.2	P = 0.32
Previous*Current	F_2,167_ = 1.2	P = 0.31	F_2,93.3_ = 0.13	P = 0.87	F_2,135_ = 0	P = 0.99	F_2,178_ = 0.5	P = 0.62
ExpTime*Prev*Curr	F_2,322_ = 0.7	P = 0.51	F_2,173_ = 0.21	P = 0.81	F_2,164_ = 1.4	P = 0.25	F_2,346_ = 0.6	P = 0.57
Ratings at time = 0	F_1,124_ = 250	P<0.001						

^#^ Analysis at times 1 and 5 minutes only, because of possible interference of previous exposure.

### Odour pleasantness

**Fig 4:**
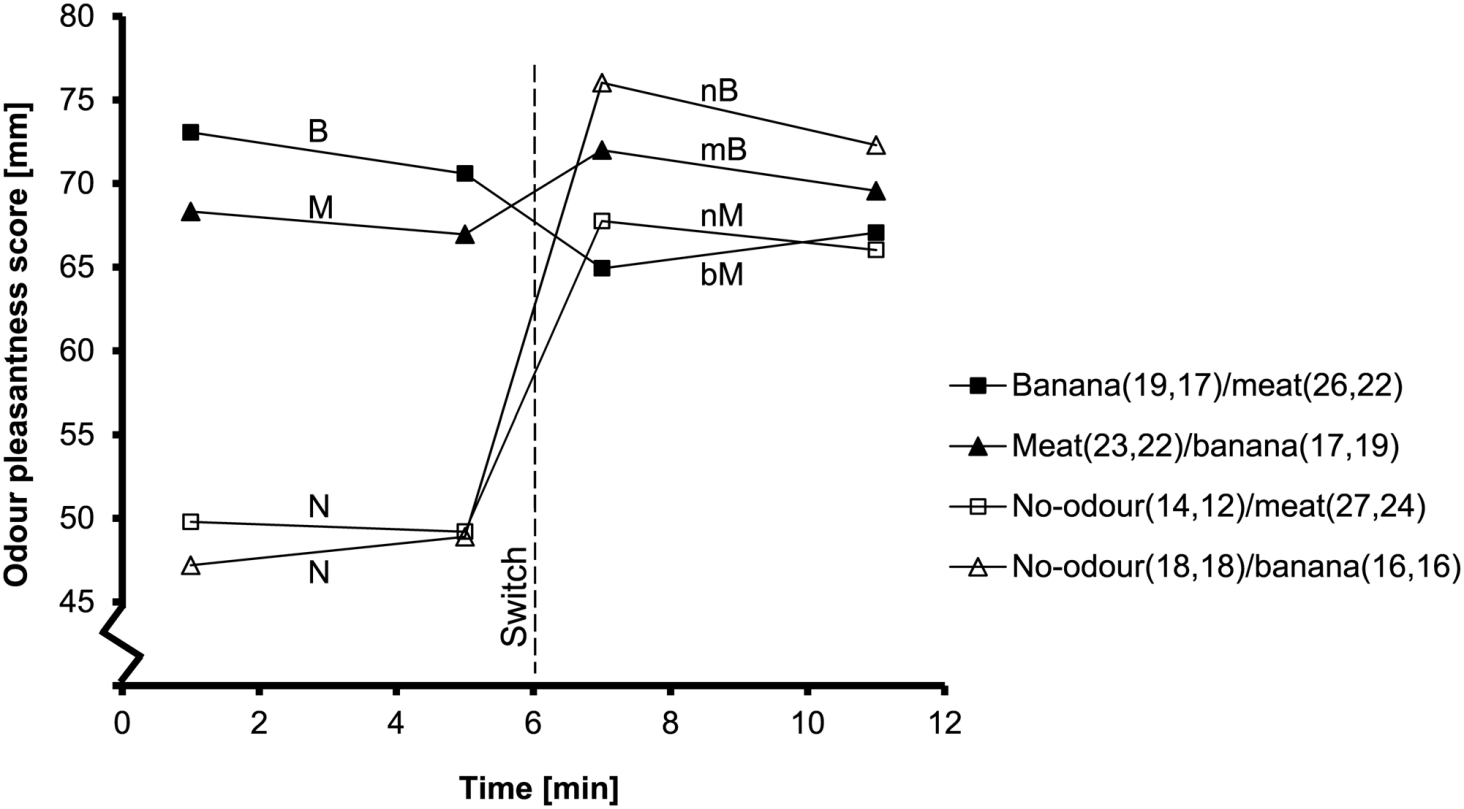
Mean odour pleasantness scores of the currently smelled odour in all combinations over time. Scores were measured on 100 mm VAS. The numbers between the brackets represent the SD at respectively 1, 5, 7 and 11 minutes. The dashed line represents the switch between odours. N: exposure to no-odour, M: meat odour, B: banana odour, nM: meat odour after no-odour, bM: meat odour after banana odour, nB: banana odour after no-odour, mB: banana odour after meat odour.

Fig 4 shows the pleasantness of the currently smelled odour. There was a significant interaction between current odour and exposure time (F_2,152_ = 3.5; P = 0.033; Table 3). Ratings decreased on average by 4 mm from 1 min to 5 min exposure time during exposure to meat and banana odour (P = 0.005). No effect of exposure time was found during exposure to no-odour (P = 0.61). Previous exposure had no significant effect on rated odour pleasantness (F_4,68.4_ = 0.3; P = 0.88). The pleasantness ratings of meat and banana odours were higher than of no-odour (both P<0.001). Differences between banana and meat odour were borderline significant (P = 0.08). Banana and meat odour ratings at 1+5 minutes were similar to ratings at 7+11 minutes (P = 0.81).

### Odour intensity

Rated odour intensity was on average 77 mm for banana, 81 mm for meat and 15 mm for no-odour. The meat and banana odours decreased in intensity by 13 mm, averaged over both periods of 1 min to 5 min exposure and 7 min to 11 min (P<0.001; Table 3). Previous exposure did not affect intensity ratings (F_4,91.5_ = 0.7; P = 0.69). The intensities of banana and meat odours were rated as higher than the intensity in no-odour (both P<0.001). Meat odour was rated 4 mm more intense than the banana odour (P = 0.015). Banana and meat odour ratings at 1 and 5 min did not significantly differ from ratings at 7 and 11 min (B vs nB and M vs nM; P = 0.20).

### Feeling well

Feeling well ratings were on average 78 mm for banana, 79 mm for meat and 74 mm for no-odour. Feeling well was rated higher during exposure to banana or meat odour than during no-odour (P = 0.013 and P<0.001 respectively). There were no differences between banana and meat odour (P = 0.11).

## Discussion

The objective of the present study was to investigate how switching between sweet and savoury odours affects the appetite for sweet and savoury products, food preference and general appetite. The results showed that the appetite for specific products adjusted within one minute to the currently smelled odour after a switch, with no lingering effect of a previously smelled odour. Apparently, our food preference system adapts within one minute to environmental changes (Fig 3). Interestingly, the pleasantness of the smelled odour decreased over time during smelling (Fig 4), whereas the appetite for the smelled food remained elevated (Fig 3).

The present appetite and preference ratings for banana, sweet, meat, savoury and staple products followed the typical pattern as found during odour exposure in previous studies [17,18], regardless of switch. This pattern comprises the odour-induced increase in the appetite and preference for congruent foods and a decrease for incongruent foods. The increase in appetite or preference for smelled foods was greater than the increase for other congruent foods [17,18]. The present results, however, display a few exceptions to this pattern. The food preferences, measured at the end of the experiment with the FPQ, shifted towards products that are congruent with the last smelled odour (Table 2), but these preferences were less pronounced after exposure to the meat/banana combination than after no-odour/banana. Possibly, the effect of the preceding meat odour on food preference slightly interfered with the effect of the banana odour, although there was no significant effect of previous exposure. Another deviation from the typical pattern is the lack of increase in the appetite for congruent sweet or savoury foods (VAS; Fig 3). In the present study, a different selection of sweet and savoury products was chosen in the appetite questionnaire than in our previous studies [17,18], which may have affected the results. Possibly not all savoury foods are congruent with the meat odour and not all sweet foods with the banana odour. Congruency with an odour may be a graded scale, with some products more congruent than others, depending on the associations of the odour with the products. Nevertheless, the appetites for the incongruent sweet and savoury foods consistently decreased during odour exposure in all studies [17,18]. Therefore, it may be concluded that sweet products are evidently incongruent with savoury odours, however, the level of congruency within a sweet/savoury category may vary, i.e. savoury products can be more or less congruent with savoury odours depending on characteristics other than odour.

Interestingly, the present results revealed a decrease in odour pleasantness over time during smelling, even though the appetite for the smelled food remained elevated. Rolls and Rolls [30] ascribed the decrease in odour pleasantness that was found in their study to olfactory sensory-specific satiety (olfactory SSS). Sensory-specific satiety is described as the decrease in pleasantness of, or desire-to-eat recently consumed foods, relative to uneaten foods [9,10]. Also the lower intake of a similar food compared with a dissimilar food, after consumption of a preload, has been attributed to SSS [10]. The name olfactory SSS suggests a lack of appetite specifically for the smelled food, whereas our present and previous [17,18] results showed an increase in the appetite for the smelled food during odour exposure, coined sensory-specific appetite (SSA) [17]. These seemingly contradictory results are perhaps the consequence of different underlying processes that determine odour pleasantness and the appetite for the smelled food. Smelling foods initiates anticipation of food intake [22] and this anticipation may consequently elevate the appetite for the smelled food, as found in our present and previous studies [17,18]. On the other hand, the decrease in pleasantness ratings during odour exposure is possibly caused by the actual stimulation of the chemical senses. Exposure to odours decreased the pleasantness of the odour that was perceived, but not the pleasantness of the taste of the smelled food that was not actually stimulated [30]. Repeatedly imagining eating M&M’s decreased subsequent food intake, but had no effect on the pleasantness of the M&M’s which indeed were not actually perceived by the senses [31]. During eating, the pleasantness of the odour and taste of the food decreases [9,30]. Therefore, we hypothesise that actual stimulation of our senses, i.e. taste buds and olfactory receptor cells, causes a decline in the pleasantness of the perceived odour and taste. The present results indicate that the decrease in odour pleasantness during exposure underlies a different construct than the changes in the appetite for specific products, although until now both observations have been explained by the opposing terms SSS and SSA.

In the present study, participants were consciously aware of the odours presented, and appetite was rated explicitly. It is also argued that olfactory information processing seems to have a strong non-conscious aspect [32,33], and can impact (eating) behaviour without awareness [34]. For instance, recent studies have shown that non-attentively perceived odours may alter subsequent food choices [19,35,36], while this was not seen when using more intense, supra-threshold odours [37]. Moreover, explicit responses can be ‘biased’ due to cognitive control, and implicit responses are sometimes suggested to better reflect participants’ intentions or behaviours, in particular when it comes to affective responses [38] and complex eating behaviours [39,40]. Though our current and previous results [17,18] indicate a clear influence of odour exposure on appetite responses, future research should also look beyond these explicit, self-reported ratings, and study implicit responses. For instance, neuroimaging techniques (fMRI [40], EEG [41]) or physiological responses of the autonomic nervous system [42] could help to better understand the mechanisms underlying and leading up to eating behaviour.

The banana and meat odour were rated as pleasant, even after the small decrease, and therefore smelling them probably still contributed to the enhancement of the appetite for those foods. The 4 mm decrease in odour pleasantness found during smelling in the present study was smaller than the 12 mm decrease that was found by Rolls and Rolls [30]. However, Rolls and Rolls [30] rated the pleasantness prior to, and after 5 minutes of smelling, whereas our participants started rating after 1 minute. Possibly, odour pleasantness already decreased in the first minute of odour exposure.

Moreover, food preference adjusted to a new odour within the first minute. Food preference may change even within the first seconds after a switch in odours, because it is known that a few seconds of food odour exposure already elicit cephalic phase responses [42]. The current set-up did not allow for such quick measurements, because answering the appetite questions for a set of 15 products took 1 minute. We avoided asking questions in the first minute, because we anticipated that changes would already take place within the first minute and we aimed to keep the circumstances, under which the first and the last questions were answered, the same as much as possible. Apparently, the largest changes in food preference occur within the first minute after odour exposure or odour switch, after which it appears to remain stable.

At the end of a test session, participants received either a banana or a bread roll with steamed meat. Twenty-two of the 30 participants always chose the same product, regardless of odour exposure, which is likely caused by strong initial preferences [43]. In addition, the FPQ data also showed that just 20% of the choices between sweet and savoury products shifted. Likely, odours are only able to change food choice when preference is ambiguous.

Finally, the present results do not support the suggestion that switching between odours affect general appetite. To our knowledge, this is the first study that investigated the effect of a switch between odours on general appetite or appetite for specific foods.

In conclusion, the appetite for specific products rapidly adjusts after a switch to the new odour and follows the typical pattern as found during odour exposure in previous studies. Surprisingly, there are no significant effects of previous exposure to odours. Interestingly, the pleasantness of the smelled odour decreases over time, whereas the appetite for the smelled food remains elevated during smelling. This seeming contradiction may result from different mechanisms, such as a decrease in hedonic value during prolonged sensory stimulation versus anticipation of food intake. Possibly, a gradual shift in food preference can be observed in the first minute after a switch between odours, when the set-up would allow for such quick measurements.

## Supporting Information

S1 File All data belonging to the study that is presented in the present paper. (XLSX)
